# Importance of proteins and mitochondrial changes as freshness indicators in fish muscle post‐mortem

**DOI:** 10.1002/jsfa.14044

**Published:** 2024-11-30

**Authors:** Nima Hematyar, Tomas Policar, Turid Rustad

**Affiliations:** ^1^ Research Institute of Fish Culture and Hydrobiology Zátiší Czech Republic; ^2^ Department of Biotechnology and Food Science Norwegian University of Science and Technology Trondheim Norway

**Keywords:** post‐mortem, protein oxidation, mitochondrial changes, myofibrillar protein

## Abstract

Evaluating protein and mitochondrial alterations post‐mortem can contribute to determining correlations between fish‐processing parameters and ultimate fish muscle quality. The myofibrillar protein alteration during rigor mortis directly affects the texture of fish muscle. To identify the mechanisms behind post‐mortem softness and quality deterioration, it is crucial to understand the conditions linked to the breakdown of myofibrillar proteins in fish skeletal muscle. Therefore, monitoring protein breakdown at the molecular level and finding target proteins would be considered a marker for fish freshness. Mitochondria play an important role in executing and regulating cell death processes, including apoptosis and necrosis. The mitochondria are the seat of cellular respiration and experience significant alterations in post‐mortem tissues. Processes used to reduce protein degradation, such as optimizing chilling and handling practices, would also minimize mitochondrial changes in fillet quality. Moreover, pH fluctuations are considered a critical point that influences both protein and mitochondrial changes. This review considered the implications of protein and mitochondrial alteration during post‐mortem storage in fish fillets and the possible pathways of their interaction on fillet quality. Mitochondrial characteristics, such as membrane integrity, pH, and ATP levels, are important for post‐mortem muscle cell changes, serving as an early indicator of fish freshness. Understanding the mechanisms behind protein degradation in fish muscle led to maintaining fillet quality and requires further experiments. Label‐free proteomics combined with bioinformatics is crucial for comprehending protein degradation mechanisms to provide customers with safe and fresh fish products while minimizing economic losses associated with fillet deterioration. © 2024 The Author(s). *Journal of the Science of Food and Agriculture* published by John Wiley & Sons Ltd on behalf of Society of Chemical Industry.

## INTRODUCTION

Because of its high content of nutrients and high absorption rate, fish has become an important animal protein in a daily diet. Together with a dramatic improvement in the resident economic level and a shift in consumption patterns,^1^ this has led to an increased demand for high‐quality fish. However, the easily accessible nutrients have negative consequences on the preservation of fish, including tissue breakdown and the simultaneous decline in quality while the fish is being stored.^2^ These characteristics include the high incidence of lipid oxidation and protein decomposition caused by microbial contamination and endogenous enzymatic activity. The fishing industry is working to enhance information on the product's date of catch to match expectations about freshness.^3^ However, the methods employed in the fishing industry to regularly evaluate freshness are more indicative of spoiling than of freshness. The processes and influencing mechanisms that impact the ultimate quality of muscle food can be broadly divided into extrinsic and intrinsic factors. Pre‐slaughter care, storage conditions, breeds of animals, feeding habits, packaging, and length of post‐mortem storage are examples of extrinsic influences. Energy metabolism, muscle pH, proteolysis, oxidative stress, and protein modification are examples of intrinsic factors.^4^, ^5^, ^6^

The most important component to clarify the underlying process of muscle softness among the previously listed components is the breakdown of myofibrillar proteins. It has been reported that the myofibrillar protein degradation indices are greatly influenced by the physicochemical properties, conformation, intermolecular force, and functional characteristics.^7^, ^8^ It has been indicated that oxidation can increase the surface hydrophobicity of myofibrillar protein by altering an amino acid and reducing its attraction to water and volatile chemicals.^9^ This suggests a close relationship between the increase in myofibrillar protein surface hydrophobicity during storage and the decline in textural parameters and water‐holding capacity (WHC).

Several studies have indicated that myofibrillar proteins change in the post‐mortem period.^10^, ^11^ Moreover, thick filament structure, particularly myosin composition, plays a critical role in determining the functional properties of myofibrillar proteins such as solubility, emulsifying, and gelling capabilities.^12^ Previous research on fish myofibrillar proteins investigated myosin and actin alterations; the most plausible reason was reported to be that persistent connections between two proteins form, which prevent muscles from freely contracting and stretching, ultimately leading to the weakening of muscles during storage.^10^, ^13^ Additionally, it was revealed that the breakdown of titin and desmin is the main way that important myofibrillar proteins contribute to softening during subsequent storage.^13^

Mitochondria are the first organelles affected by tissue anoxia in post‐mortem skeletal muscle cells. Damage to mitochondria occurs in post‐mortem skeletal muscle due to a decrease in antioxidant defense and an increase in the generation of reactive oxygen species (ROS).^14^, ^15^ Several studies have indicated the role of mitochondria on muscle quality during post‐mortem storage,^15^, ^16^ and that in bovine, marine, and human models mitochondrial activity was still detectable after death during storage at +4 °C.^3^, ^17^

Structural alterations in post‐mortem mitochondria have been linked to alterations in myofibrillar protein structure.^18^, ^19^ Investigation by electron microscopy has documented post‐mortem morphological mitochondrial alterations, including edema and broken cristae, in muscle from Pacific bluefin tuna (*Thunnus orientalis*) and gilthead sea bream (*Sparus aurata*) stored under cold conditions.^18^, ^20^

This review investigates the importance of proteins and mitochondrial alterations on fish muscle quality and the possibility of introducing them as markers of freshness during post‐mortem storage.

## TARGET PROTEINS IN FISH MUSCLE POST‐MORTEM

**Figure 1:**
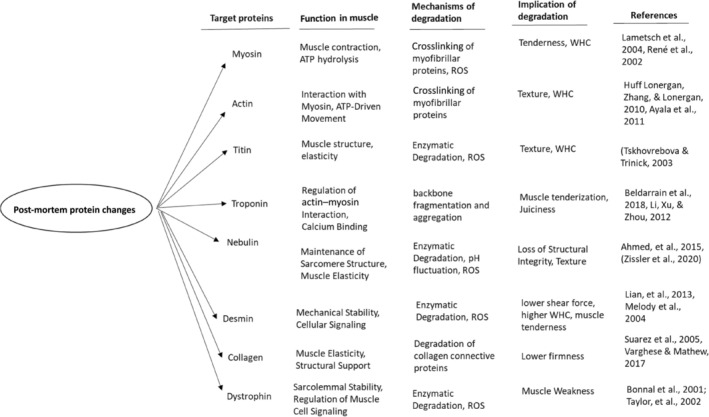
Implications of protein degradation for target proteins in Post‐mortem fish muscle ^18^ ^,^ ^28^ ^,^ ^30^ ^,^ ^38^ ^,^ ^41^ ^,^ ^51^ ^,^ ^52^ ^,^ ^98^ ^,^ ^99^ ^,^ ^100^ ^,^ ^101^ ^,^ ^102^ ^,^ ^103^.

Proteins have crucial roles in fish muscle structure and are important for WHC, texture, hydrophobicity, and solubility of proteins.^21^, ^22^ The protein functionality is changed post‐mortem and, subsequently, freshness as well as final muscle quality parameters might be impacted (Fig. 1).

Due to impaired antioxidant defense mechanisms, ROS are not effectively removed from the body after animal death. The degree of early protein oxidation has been detected in aquatic product muscle tissue and varies according to the animal species, age, and type of muscle.^23^ For instance, the initial content of carbonyls in silver carp (*Hypophthalmichthys molitrix*) was 1.42 nmol mg^−1^.^24^ Conversely, tilapia (*Oreochromis niloticus*), another freshwater fish, had an initial value of less than 1 nmol mg^−1^,^25^ indicating a lesser extent of protein carbonylation. However, it was observed that the initial carbonyl content of sea cucumber (*Stichopus japonicus*)^26^ and shrimp (*Penaeus vannamei*),^27^ was much higher (2.98 and 32.08 nmol mg^−1^, respectively).

Furthermore, owing to calpain enzyme activity, protein degradation begins early post‐mortem. Despite substantial investigations regarding protein alteration post‐mortem, some mechanisms of protein degradation are still not clear. Moreover, different fish species have been shown to have different target proteins during the post‐mortem process. In this regard, dystrophin protein in salmon and sea bass and myosin in African catfish are the most important textural markers.^28^, ^29^, ^30^ According to some reports, dystrophin can serve as an early marker for freshness in fish flesh.^28^, ^30^

Additionally, the stability of protein during post‐mortem storage can be different. It has been reported that R‐connectin as a cytoskeletal protein can be completely converted to α‐connectin after 2 days in carp, which indicates R‐connectin degradation, but in rainbow trout this process is not completed even after 4 days.^31^ Furthermore, the degradation of dystrophin has been commented on in sea bass during storage at 4 °C,^32^ which indicates total degraded dystrophin protein in 48 h after death.

**Figure 2:**
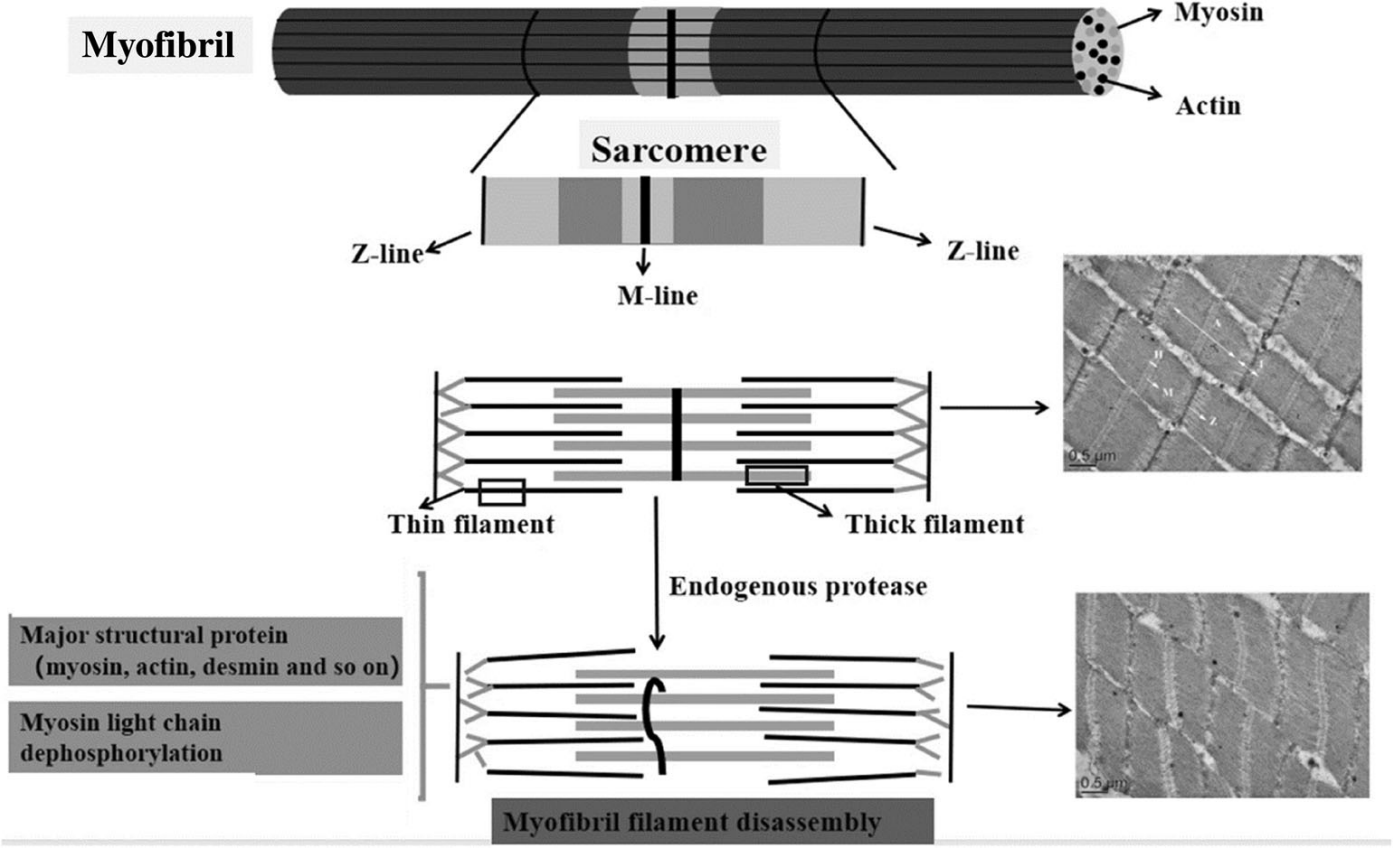
Diagram showing how endogenous protease breaks down proteins (Jiang *et al*., ^95^ Liu *et al*., ^9^ Ge *et al*.,^96^).

As touched upon above, texture, as a primary factor of muscle food, is affected by protein changes, particularly by myofibrillar protein alteration rather than connective tissue changes.^11^, ^33^ (Fig. 2).

Furthermore, within 24 h after death, fish muscles showed a detachment of myofiber to connective tissue (endomysium). The quantification of these fractures indicates that they are linked to the texture of fish fillets and likely cause most of the first texture alterations. In addition, released α‐actinin from myofibrils can be considered a good marker of disorganization in fish muscle post‐mortem. With the lack of energy, the cross‐bridge between actin and myosin forms permanently, resulting in muscle shortening. It seems that the presence of cysteine close to the myosin tail is one important reason that can reduce WHC and increase firmness through the crosslink bonds in this area.

On the other hand, due to the oxidation of myoglobin, myosin thiyl and tyrosyl free radicals are formed. The aggregation of myosin, particularly the heavy chain, is facilitated by the mentioned free radicals. These crosslinks are more prevalent in the myosin tail, especially when oxidations are mediated by metmyoglobin or iron.^34^

ATP is crucial for muscle processes as it provides the energy needed for muscle contraction. This process begins with the release of Ca^2+^ ions in the sarcoplasm after the muscle cell receives a nervous impulse to contract.^35^ Following death, muscle cells continue to metabolize glycogen through anaerobic glycolysis, leading to the accumulation of lactic acid, resulting in reduced pH.^36^ This triggers the release of calcium ions from the sarcoplasmic reticulum.^37^ In the post‐mortem period, calcium can bind actin and myosin proteins together, leading to the firmer muscle. It is plausible that restricted proteolysis of myofibrillar actin may modify the actin/myosin rigor link.

In the process of contraction, titin contributes to keeping the myofibril's sarcomeric alignment. The thick filaments (A‐band) are located in the middle of the sarcomere and preserved by titin, which integrates the Z‐line and thickness filaments. Furthermore, it is proposed that titin contributes to the production of some of the passive tension found in skeletal muscle cells.

Additionally, the thin filaments in the adjacent I‐band regions ultimately lead to a decrease in the muscle cell's structural integrity.^38^

The portion of nebulin spanning the A–I junction has been shown to inhibit actomyosin ATPase activity and sliding velocities of actin filaments over myosin.^39^ Studying the role of nebulin in post‐mortem muscle changes offers exciting opportunities to uncover novel pathways and mechanisms of muscle tenderization.

Furthermore, during muscular contraction, calcium ions are attached to the nh2 domain of troponin C, facilitating its interaction with troponin I and leading it to dissociate from actin. Consequently, the connecting proteins are eliminated with troponin degradation, and the thin filaments inside the sarcomeric I band can be broken. Overall, the degradation of troponin T and the appearance of polypeptides in the 30 kDa region indicate muscle tenderness during post‐mortem aging.

Desmin, a type III intermediate filament protein, plays a crucial role in maintaining the structural integrity of muscle cells, particularly in skeletal muscle.^40^ It is localized at the periphery of the myofibrillar Z‐disk and surrounds the Z‐lines of myofibrils, connecting adjacent myofibrils and linking them to other cellular structures, including the sarcolemma.^41^ A major degradation product of desmin, typically observed in muscle, is a polypeptide of approximately 38 kDa.^42^ μ‐Calpain, a calcium‐dependent protease, is implicated in desmin degradation under normal post‐mortem aging conditions. However, the direct relationship between desmin degradation and muscle tenderness is still under investigation. It remains to be determined whether desmin degradation directly contributes to tenderness development or serves as an indicator of overall post‐mortem proteolysis.

Post‐mortem changes in collagen include solubilization and degradation. There is a correlation between the decreasing trend in the type V collagen content and post‐mortem fish muscle softening during chilled storage,^43^ which might be related to the cleavage of collagen by crosslinks. The presence of calcium ions facilitates the formation of crosslinked bonds between collagen molecules in the muscle tissue.^44^ These crosslinks stabilize the collagen structure, making it more resistant to enzymatic degradation and mechanical disruption.

Most probably, side‐chain oxidation has less impact on the quality of proteins in the early stage of rigor mortis, and it might occur through backbone oxidation between actin and myosin.^21^ By elucidating the molecular processes underlying post‐mortem muscle degradation, researchers can develop targeted strategies to optimize muscle aging processes and enhance muscle quality. This knowledge may also have broader implications for understanding muscle function and dysfunction in various physiological and pathological conditions.

## IDENTIFICATION OF ALTERED PROTEIN BY PROTEOMIC APPROACH

**Figure 3:**
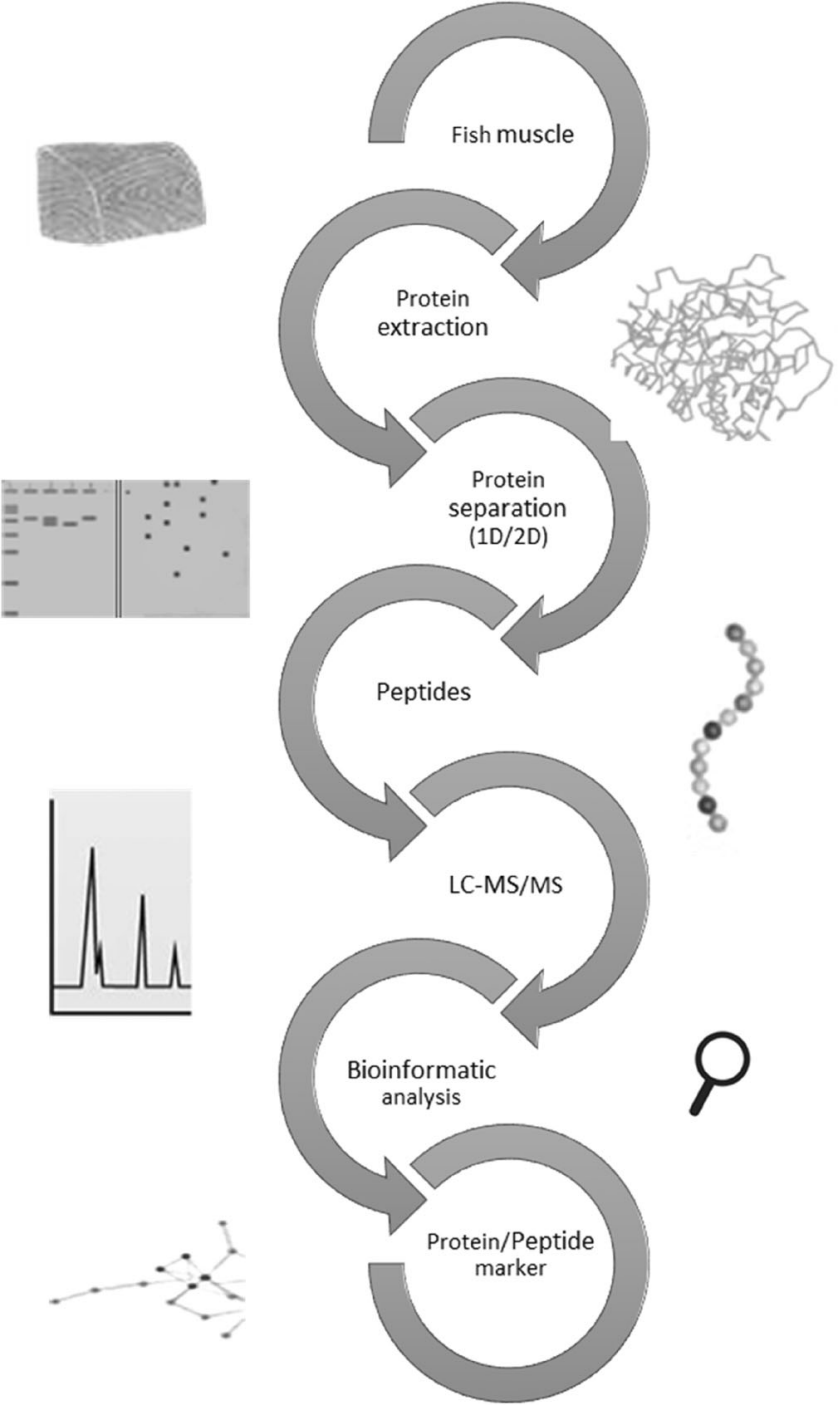
Outline of a typical proteomic experiment schematic on muscle food.

Identifying altered proteins using proteomic approaches involves a series of steps aimed at characterizing the proteins present in a biological sample and comparing their expression levels or modifications under different conditions (Fig. 3).

Expression and functional proteomics are the two primary fields of proteomics research. It is commonly recognized that the majority of biological processes are controlled by protein complexes rather than single proteins and that the interactions between these proteins may determine a given biological function.^45^ Different proteomic approaches, including mass spectrometry (MS), protein microarray, two‐dimensional gel electrophoresis (2DE), and chromatographic procedures, are employed for expression profiling.^46^ The mentioned methods have been comprehensively reviewed.^47^ Therefore, we will not discuss them further in this review.

Protein degradation has been investigated in sea bass (*Dicentrarchus labrax*) muscle and was shown to have a higher degradation in some protein spots.^48^ In addition, comparative analysis showed that seabass myosin may degrade even at low storage temperatures. Similarly, myosin heavy chain and myosin light chain breakdown has been demonstrated in myofibrillar proteins of African catfish, Eurasian perch, and signal crayfish stored at different temperatures.^11^, ^29^, ^33^ With respect to deterioration processes, proteome analysis has also been reported for shrimps.^49^ In this study, proteome analysis of Arctic (*Pandalus borealis*) and tropical shrimps (*Penaeus japonicus* and *Penaeus monodon*) showed differences in post‐mortem degradation of muscle proteins between cold‐water and warm‐water species. The degradation of myosin in *Pandalus borealis* by partial purification of this molecule by electrophoresis under native conditions for 9 h has been reported^49^ and subsequent sodium dodecyl sulfate–polyacrylamide gel electrophoresis analysis of the bands of gel containing native isomyosins. Differentially expressed proteins (arginine kinase, phosphopyruvate hydratase, and actin T2) in the Pacific white shrimp (*Litopenaeus vannamei*) muscle have been identified,^50^ which were downregulated during storage time at +4 °C Tenderization of muscle foods during the post‐mortem process has contributed to the degradation of the mentioned myofibrillar proteins.^51^ However, it is reasonable to believe that even a minor degradation of actin will weaken the myofibrillar lattice and thereby influence the texture of the meat, even though it could be expected that the degree depends on where the thin filament's actin is degraded. Seven marker proteins (heat shock protein, troponin C, creatine kinase, β1‐actin, myozenin 1, troponin T, and myosins 1 and 2) have been detected^52^ as an indicator of tenderness in meat via a liquid isoelectric focusing (OFFGEL) approach. It appears that moderate protein degradation would improve the ultimate muscle quality.

Transcriptomic analysis provides a valuable method for understanding the molecular mechanisms behind protein changes during post‐mortem time by considering protein alteration alongside genes responsible for protein expression. Integrating transcriptomic and proteomic data can provide a comprehensive understanding of the regulatory networks and biological processes governing meat quality traits and aging.

## ASSESSING SEAFOOD FRESHNESS USING MITOCHONDRIAL ACTIVITY

**Figure 4:**
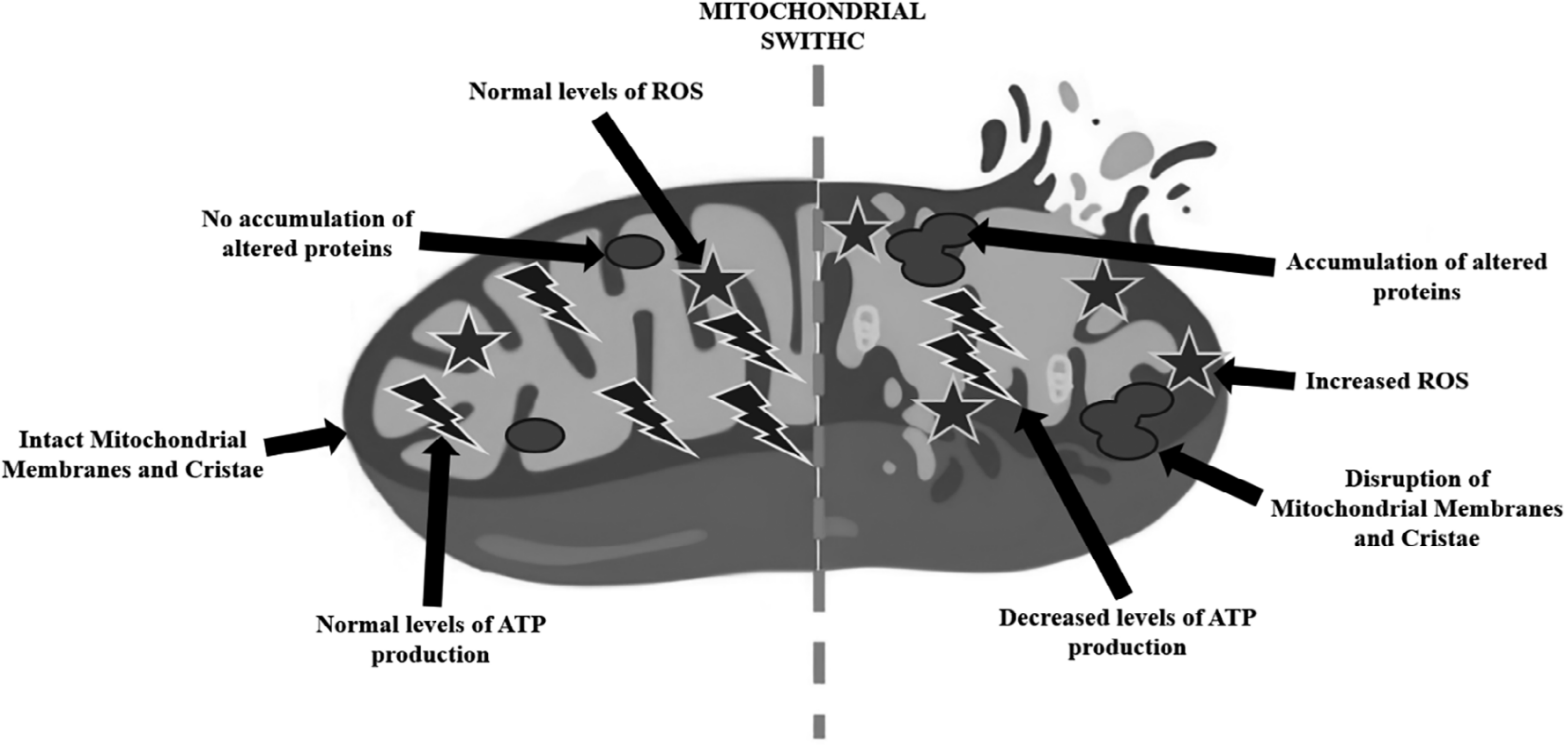
The illustration shows the appearance and characteristics of a mitochondrion before and after an injury that resulted in the organelle's dysfunction. This process leads to an increase in reactive oxygen species (ROS), causing changes in proteins, rupturing the membranes and cristae of the mitochondria, and enlarging the mitochondria (Stanga *et al*., ^97^).

Mitochondrial activity can serve as an indicator of fish freshness. As fish undergo post‐mortem changes, the activity of mitochondria can provide insights into the metabolic state of the muscle tissue and its freshness (Fig. 4). Indeed, mitochondria play a crucial role in cell death mechanisms such as apoptosis and necrosis.^19^

During apoptosis, mitochondria release pro‐apoptotic factors such as cytochrome c, which activate caspases and initiate the apoptotic cascade. In necrosis, mitochondrial dysfunction can release damage‐associated molecular patterns (DAMPs), triggering an inflammatory response and cell death.

Proteases are enzymes that naturally exist in seafood and become active after the fish or shellfish is harvested. Once activated, these enzymes start breaking down the proteins in the muscle, which causes changes in texture and eventually leads to spoilage. Caspases are enzymes involved in programmed cell death. After seafood is harvested, these enzymes contribute to muscle breakdown, affecting product quality and freshness. After seafood is harvested, the mitochondria, which are the energy source of cells, begin to break down. This breakdown causes them to swell and lose their ability to function. As a result, the process of protein breakdown speeds up, directly affecting the freshness of the seafood.

Several studies have demonstrated that mitochondrial activity continues post‐mortem in various animal models, including bovine, marine, and human tissues.^17^, ^53^

Electron microscopy studies have documented post‐mortem morphological mitochondrial alterations, including swelling and damaged cristae in muscle from Pacific bluefin tuna (*Thunnus orientalis*) and gilthead sea bream (*Sparus aurata*) under cold storage. It was reported^18^, ^20^, ^54^ that the alterations in mitochondria are sensitive to the length of storage, suggesting that the modifications in mitochondria occurred concurrently with the changes in myofibrillar structural proteins,^15^ suggesting a close relationship between mitochondria and tenderness.

As touched upon above, an increase in storage duration was correlated with an increase in mitochondrial swelling.^54^ Enhanced swelling in mitochondria can occur due to various factors that disrupt mitochondrial function and integrity.

Increased production of ROS can damage mitochondrial membranes and proteins, leading to mitochondrial swelling. ROS‐induced lipid peroxidation and oxidative damage compromise membrane integrity and disrupt ion homeostasis, promoting mitochondrial swelling.^55^ Moreover, oxidative stress causes the depolarization of mitochondrial potential, swelling, and opening of the mitochondrial membrane. This increased caspase‐3 activation and cytochrome c release from mitochondria.^56^ Eventually, oxidative stress caused myofibril sensitivity to proteolysis and led to the breakdown of some proteins (desmin and troponin T).^57^

Additionally, excessive influx of calcium ions into mitochondria can trigger mitochondrial swelling by activating calcium‐dependent enzymes, such as the mitochondrial permeability transition pore (MPTP).^58^ Opening of the MPTP leads to osmotic imbalance, water influx, and mitochondrial swelling. Calcium overload can trigger the activation of calcium‐dependent proteases, such as calpains and caspases, within the mitochondrial matrix.^59^ These proteases are involved in the degradation of specific mitochondrial proteins and regulatory factors, leading to alterations in protein turnover and cellular homeostasis. The apoptotic pathway is initiated by activated calpain I and cathepsins.^14^ The mitochondrial apoptotic proteins are closely linked to these enzymes, which also generate signaling molecules that trigger the caspase system. Calpain I auto‐cleaves and becomes active in cells when it comes into contact with Ca^2+^.^24^ Consequences of Ca^2+^ accumulation during post‐mortem time are linked to the protease enzyme activities. Ultimately, this pathway contributes to facilitating the degradation of myofibrillar proteins.

Moreover, dysregulation of the MPTP, either by prolonged calcium overload or other factors, can result in the uncontrolled opening of the pore, leading to mitochondrial swelling.^15^ This process is associated with mitochondrial dysfunction and cell death pathways. Disruption of the mitochondrial outer membrane and release of pro‐apoptotic proteins, such as cytochrome C, can trigger apoptotic pathways and promote mitochondrial swelling. Cytochrome C release activates caspases and initiates apoptotic cell death.

Overall, enhanced swelling in mitochondria can result from a complex interplay of cellular stressors, including oxidative damage, calcium dysregulation, membrane permeability changes, and apoptotic signaling pathways. Understanding the mechanisms underlying mitochondrial swelling is crucial for elucidating its role in disease pathogenesis and identifying potential therapeutic targets for mitochondrial dysfunction‐related disorders. Caspase activation during apoptosis can lead to the cleavage of structural proteins in muscle tissue, such as actin, myosin, troponin, and desmin. Cleavage of these proteins disrupts the cytoskeletal structure and contractile apparatus of muscle fibers, contributing to tissue degradation. Liquid chromatography–tandem mass spectrometry (LC‐MS/MS) was utilized^60^ to track proteomic changes in large yellow croaker (*Pseudosciaena crocea*) muscle during storage. Results indicated a significant decrease in mitochondrial proteins, such as ATP synthase and cytochrome c oxidase, as the storage time increased. Simultaneously, there was an observed increase in the degradation of structural proteins, highlighting a clear connection between mitochondrial dysfunction and protein degradation. The freshness of tail and claw muscles in signal crayfish (*Pacifastacus leniusculus*) was investigated using a proteomics approach. It was indicated that there is a correlation between protein and ATP degradation as freshness indicators during storage time. A decrease in mitochondrial proteins and the breakdown of myofibrillar proteins during the storage of hake (*Merluccius merluccius*) fillets were revealed by the proteomics approach.^11^, ^61^ This simultaneous change strongly suggests a correlation between mitochondrial dysfunction and overall protein degradation, serving as indicators of freshness. The decline of mitochondrial proteins and the increase in protein degradation observed through proteomics studies show a clear correlation. This approach is essential for ensuring the freshness and safety of seafood products, providing valuable insights into seafood quality assessment.

## PROMISING APPROACHES TO INTRODUCE MITOCHONDRIAL ACTIVITY AS A FRESHNESS INDICATOR

The lack of standardized methods for measuring mitochondrial dysfunction would be a challenge for future research. Most of the studies on mitochondrial changes in seafood use indirect indicators like ATP depletion, lipid oxidation, or changes in mitochondrial membrane potential. However, these methods vary across studies, making it difficult to compare findings. To address this issue, a standardized protocol for assessing mitochondrial damage could be developed, involving high‐resolution respirometry to measure oxygen consumption rates or the use of fluorescent dyes to quantify mitochondrial membrane potential. This would help ensure consistent and comparable data across studies.

Quantifying cellular respiration is a keyway to assessing mitochondrial function because it involves many sequential events, from glycolysis to oxidative phosphorylation.^62^ It also immediately indicates any problems with the electron transport chain.^63^ Therefore, we can assess the capacity to produce ATP and measure mitochondrial activity by estimating oxygen consumption rates (OCR). The mitochondria are the primary organelles that consume oxygen and generate ROS.^55^ Oxidative stress occurs when cells are unable to metabolize ROS intermediates. Therefore, using the Seahorse XFp Analyzer to conduct a functional investigation could shed light on the role of mitochondrial dysfunction.^64^, ^65^

The mitochondrial proteome has been largely used in studies, despite the extensive use of proteomics to investigate protein breakdown in seafood. It is currently unknown how mitochondrial proteins behave during post‐mortem storage and what role they play in spoilage. Conducting proteomic research that specifically focuses on mitochondrial proteins may lead to the discovery of potential freshness indicators. Advanced proteomic techniques, such as LC‐MS/MS and 2D gel electrophoresis, are used to identify and quantify mitochondrial proteins in seafood, enabling the detection of changes in protein expression and post‐translational modifications.^66^, ^67^ Additionally, isobaric tags for relative and absolute quantitation (iTRAQ) are used to study mitochondrial changes in seafood by profiling complex proteomes and identifying biomarkers for oxidative stress and freshness deterioration. It allows for the simultaneous identification and quantification of proteins (cytochrome c oxidase or ATP synthase), particularly useful in monitoring mitochondrial dysfunction linked to seafood spoilage.^68^, ^69^ iTRAQ is used to analyze changes in the abundance of mitochondrial proteins in fish muscle tissue during chilling, freezing, or antioxidant treatment, indicating mitochondrial dysfunction that affects seafood quality.

Furthermore, while mitochondrial dysfunction often involves lipid peroxidation, few studies have explored this aspect in seafood freshness. Understanding how lipid oxidation within mitochondria contributes to spoilage could uncover new freshness indicators. This peroxidation can lead to mitochondrial dysfunction, speeding up the production of ROS and worsening oxidative stress in muscle tissues.^70^, ^71^ It appears that there is a clear link between lipid oxidation, damage to mitochondrial membranes, and the deterioration of quality during storage. These findings indicate that future research should prioritize the mitochondrial lipidome in seafood and explore how lipid peroxidation (malondialdehyde or 4‐hydroxynonenal) could act as an early indicator of freshness, in addition to protein degradation.

## CONTROL OF MITOCHONDRIAL CHANGES DURING POST‐MORTEM STORAGE OF FISH

For a while after death, mitochondria in post‐mortem muscle retain their structural integrity and proceed to use oxygen, which influences the myoglobin level and oxidative stress. By encouraging the deterioration of cytoskeletal and myofibrillar proteins, mitochondria‐mediated apoptosis also affects the process of post‐mortem muscle tenderization.^15^ Therefore, through their effects on oxidative stress, modifications in the myoglobin redox status, glycolysis, and apoptosis, mitochondria have an impact on the production of meat color, WHC, tenderness, and taste.

Minimizing mitochondrial changes is essential for retaining the quality and freshness of fish products. Immediately after harvest, fish should be cooled rapidly to slow down metabolic processes and preserve mitochondrial integrity. Rapid cooling inhibits enzymatic activity and reduces the rate of mitochondrial degradation.^72^ Exploring the interplay between mitochondrial function, sarcoplasmic reticulum integrity, and storage conditions is crucial for enhancing seafood quality. The storage temperature has a significant impact on the functions of both mitochondria and the sarcoplasmic reticulum. Low temperatures can help maintain mitochondrial activity and prevent rapid deterioration of seafood quality. However, fluctuating temperatures can worsen mitochondrial dysfunction and stress on the sarcoplasmic reticulum, which can lead to a decline in seafood freshness.^73^, ^74^ It appears that temperature has a significant impact on the activity of mitochondria in seafood, affecting metabolic processes and overall quality. Mitochondria, which are responsible for energy production through oxidative phosphorylation, are sensitive to temperature changes. Higher temperatures can impair mitochondrial function, leading to decreased rates of oxygen consumption and changes in membrane fluidity.

Applying antioxidants, such as ascorbic acid or tocopherols, can help mitigate oxidative stress and preserve mitochondrial function during post‐mortem storage. Antioxidants scavenge free radicals and protect mitochondrial membranes from damage.^75^

Modified atmosphere packaging involves packaging fish with modified oxygen and carbon dioxide levels in a controlled atmosphere. This technique can help preserve mitochondrial changes by reducing oxidative stress and delaying the onset of rigor mortis.

Controlling the pH in fish muscle during post‐mortem is crucial for preserving mitochondrial function and minimizing protein degradation. Rapid pH decline post‐mortem can lead to mitochondrial dysfunction and proteolysis, resulting in apoptosis, caspase‐3 activation and, ultimately, reduced muscle firmness.^76^ Since practically all cellular activities depend on a constant pH, cytosolic pH is a physiological parameter that is strictly regulated in all cellular systems. Protein folding, signal transduction, metabolism, and protein–lipid interactions can all be significantly impacted by small variations in cytosolic pH.^77^, ^78^ The cytosolic pH changes in fish muscle are caused by various stressors such as stock density, stunning methods, and storage conditions.^79^, ^80^ Intracellular pH fluctuations have a substantial correlation with post‐mortem followed by protein and mitochondrial changes.

Managing pH changes through appropriate chilling techniques and additives can help preserve mitochondrial integrity. Further investigation is required to confirm the impacts of apoptosis, oxidative stress, and energy metabolism on fish fillet quality.

Mitochondrial changes could influence post‐mortem protein degradation and mitochondria as an indicator of fish freshness need more consideration.

## ROLE OF pH ON PROTEIN AND MITOCHONDRIAL CHANGES

Owing to differences in fish farming and harvest conditions, changes are a complex phenomenon and different alteration targets and pathways might be expected. Muscle pH seems to have a crucial impact on the ultimate muscle quality. Both protein and mitochondrial changes, suggested as freshness indicators, are influenced by pH. Important factors influencing protein oxidation include feeding, pre‐mortem management, preservation, and processing. Therefore, fish welfare might be studied in more depth, as all the welfare parameters can affect pH and subsequently protein and mitochondrial alterations.^81^

pH has a significant impact on proteolytic enzyme activities, subsequently affecting the aging potential of muscle texture. In post‐mortem muscle, the combination of inadequate ATP production and reduced pH disrupts the normal redox system, leading to the accumulation of pro‐apoptotic free radicals.^82^, ^83^ Higher levels of ROS directly correspond to lower muscle pH.^84^ Antioxidant enzymes like superoxide dismutase, catalase, and glutathione peroxidase were found to have reduced activity in post‐mortem bovine muscle at low pH, as previous studies have indicated.^85^ Post‐mortem fast pH decline resulted in sarcoplasmic protein denaturation and mitochondrial dysfunction, contributing to the accumulation of muscle ROS. A low pH led to reduced degradation of titin and increased degradation of nebulin post‐mortem, compared to a high pH in muscle.^86^

Tender meat has been consistently observed to undergo faster and more extensive degradation of desmin, troponin T, nebulin, and titin when compared to tough meat. Scientists have made efforts to determine a suitable duration of aging to prevent softening by monitoring the degradation of these proteins.^87^, ^88^

The degradation of troponin T during post‐mortem aging primarily results in products of approximately 30 kDa. For example, it was reported that at 24 h post‐mortem the 30 kDa band is present in tender bovine muscle and yet is not detected in tough bovine muscle.^51^ According to the study, the degradation of troponin T in high‐pHu (ultimate pH) beef was initiated right after slaughter, resulting in the development of a 30 kDa product. The degradation protein pathway in the low‐pHu group seems to be similar to the high‐pHu group in muscle.^89^ The breakdown of troponin T in intermediate‐pHu beef was significantly slower; only minor degradation was observed within 24 h post‐mortem, and intact troponin T hardly disappeared until day 3 post‐mortem.^89^ These results are in line with another study,^90^ which reported that troponin T breakdown products started to become apparent 2–3 days after death.

Since desmin is a component of the costameres and intermediate filaments that bind myofibrils to the sarcolemma and connect myofibrils at the Z‐disk level, respectively, it plays a significant role in the ultrastructure of muscle.^91^ We proposed that the rate and extent of myofibrillar protein degradation could be different based on the muscle pHu. The post‐mortem degradation of desmin was found to be faster in high‐pHu muscle, and the slowest in intermediate‐pHu muscle.^89^ The variations in calpains and cathepsin B activity at the extremes of post‐mortem muscle pH may explain the observed occurrence.

Muscle exhibiting a rapid decline in pH also showed greater mitochondrial membrane permeability.^92^ This suggests a potential correlation between pH decline and mitochondrial function, with lower pH possibly leading to alterations in mitochondrial membrane integrity. As each fish species showed different target protein oxidation post‐mortem, considering the changes in pH can provide a better overview of the final fillet quality. Additionally, it was reported that under acidic conditions (pH 5.6) there was a reduction in mitochondrial metabolic activity compared to neutral pH (pH 7.2).^53^ This reduction in metabolic activity indicates a potential impairment of mitochondrial function in acidic environments, possibly due to increased mitochondrial degradation or other mechanisms. Excessive calcium release from mitochondria, coupled with acidic conditions, can lead to mitochondrial dysfunction.^93^ This dysfunction can disrupt the inner mitochondrial membrane. As soon as mitochondrial membrane integrity is compromised, calcium stored within the mitochondria is released into the cytoplasm. This calcium influx might activate calpain‐1, a calcium‐dependent protease.^94^ Calpain‐1 activation might result in the degradation of specific cellular proteins, such as troponin T and desmin. These proteins are important for muscle function and structure.

## CONCLUSIONS

Due to the degradation of proteins and the formation of smaller peptides and free amino acids, fish muscle becomes softer. Developing new and reliable methods is necessary to investigate and find a correlation between post‐mortem changes and protein alteration as critical markers during rigor. Proteomics might be used to build biosensors and protein arrays for rapid field testing, as well as to discover proteins that indicate certain muscle food quality and enhance food quality in fisheries. Food quality is improved by this technology, which enables fisheries and food control agencies to do testing at their facilities without the need for costly equipment or trained personnel.

Necrosis in fish mitochondrial tissue is a type of cell death that can have significant implications for the quality of fish muscle and our understanding of post‐mortem changes. Therefore, optimizing handling and processing, as well as considering early biomarker detection, would help maintain fillet quality for a longer time.

In summary, during the post‐mortem period, proteins and mitochondrial alterations are significant markers of freshness in fish muscle. Monitoring these changes allows for assessing fish quality and helps prevent the sale or consumption of spoiled fish products.

## AUTHOR CONTRIBUTIONS

Conceptualization: Nima Hematyar. Funding acquisition: Tomas Policar. Supervision: Turid Rustad. Writing – original draft: Nima Hematyar. Writing – review and editing: Nima Hematyar, Turid Rustad, and Tomas Policar. All authors have read and agreed to the published version of the manuscript. All authors reviewed the manuscript.

## CONFLICT OF INTEREST

The authors declare no real or perceived conflicts of interest. The authors declare that they have no known competing financial interests or personal relationships that could have appeared to influence the work reported in this paper.

## Data Availability

The data that support the findings of this study are available on request from the corresponding author. The data are not publicly available due to privacy or ethical restrictions.
